# Manufacturing Order Matters? Exploring the Impact of Heat Treatment and Machining Sequences on the NiTi Instruments Properties

**DOI:** 10.1111/aej.12953

**Published:** 2025-05-26

**Authors:** Emmanuel J. N. L. Silva, Jorge N. R. Martins, Livia Neri Menezes de Oliveira, Mylena do Rosário do Pereira, Murilo Priori Alcalde, Victor T. L. Vieira, Francisco Manuel Braz Fernandes, Marco A. Versiani

**Affiliations:** ^1^ School of Dentistry Grande Rio University (UNIGRANRIO) Rio de Janeiro Brazil; ^2^ Department of Endodontics Rio de Janeiro State University Rio de Janeiro Brazil; ^3^ Deparment of Endodontics Fluminense Federal University Niterói Brazil; ^4^ Faculdade de Medicina Dentária Universidade de Lisboa Lisboa Portugal; ^5^ Grupo de Investigação Em Bioquimica e Biologia Oral, Unidade de Investigação em Ciências Orais e Biomédicas (UICOB), Faculdade de Medicina Dentária Universidade de Lisboa Lisboa Portugal; ^6^ Centro de Estudo de Medicina Dentária Baseada na Evidência (CEMDBE) ‐ Cochrane Portugal, Faculdade de Medicina Dentária Universidade de Lisboa Lisboa Portugal; ^7^ Department of Endodontics, Bauru Dental School São Paulo University Bauru Brazil; ^8^ Department of Materials Science, CENIMAT/I3N, NOVA School of Science and Technology Universidade NOVA de Lisboa Caparica Portugal; ^9^ Dental Specialty Center Brazilian Military Police Belo Horizonte Minas Gerais Brazil

**Keywords:** differential scanning calorimetry, geometric design, heat treatment, mechanical performance, metallurgy, NiTi instruments

## Abstract

This study assessed how manufacturing sequence affects the metallurgical and mechanical properties of two nickel‐titanium instruments: Procodile Q (heat‐treated before machining) and Procodile +HT (heat‐treated after machining). Both instruments (*n* = 74 each, size 25/0.06) were analysed for design, NiTi composition, phase transformation temperatures and mechanical performance (cyclic fatigue at 20°C and 35°C, torsional fatigue, bending load, buckling strength, cutting ability, microhardness and roughness). The Student's t‐test and Mann–Whitney test were used for statistical analysis (*α* = 5%). Results showed that both instruments had similar design and composition. Procodile Q had a slightly higher austenitic start temperature and superior cyclic fatigue resistance at 20°C and 35°C (*p* < 0.001), along with higher surface roughness (*p* = 0.041). Other mechanical properties showed no significant differences (*p* > 0.05). These findings suggest that the order of machining and heat treatment of Procodile instruments had minimal impact on overall performance, primarily affecting cyclic fatigue resistance and surface roughness.

## 1 Introduction

The introduction of nickel‐titanium (NiTi) rotary instruments has marked a significant advancement in endodontics, transforming root canal shaping procedures and contributing to more favourable clinical outcomes [1]. Recent metallurgical innovations have led to the development of a wide variety of NiTi instruments, differing in kinematics, surface treatments and design [2, 3]. Despite these advancements, NiTi instruments remain susceptible to fractures, particularly in challenging clinical scenarios such as curved and/or calcified root canals. These vulnerabilities can compromise treatment success, potentially leading to persistent apical periodontitis and treatment failure [4, 5]. An important development in the production of NiTi instruments has been the application of various heat treatments aimed at enhancing their mechanical properties and clinical performance. These heat treatments involve heating the NiTi alloy to precise temperatures followed by controlled cooling, which induces the formation of a martensitic structure at room temperature. This specific crystalline phase significantly improves the flexibility and resistance to cyclic fatigue of the instruments, reducing the likelihood of fractures [6]. As a result, in recent years, nearly all manufacturers have incorporated heat‐treated NiTi instruments into their product lines, providing clinicians with a valuable alternative to instruments made from conventional NiTi alloys. This shift reflects the growing recognition of the benefits that heat treatment can bring to instrument performance. Consequently, continued research and development focused on optimising the manufacturing processes of NiTi instruments is crucial for enhancing their reliability, durability and overall clinical performance.

While heat treatments have unquestionably enhanced the properties of NiTi instruments, the specific sequence of manufacturing steps, particularly the order in which heat treatment and machining processes are applied, may play an important role in determining the final metallurgical and mechanical characteristics of these instruments. Heat treatment, through controlled heating and cooling cycles, can increase flexibility and fatigue strength by stabilising martensitic or R‐phase crystallographic arrangements, while machining processes such as grinding or milling may introduce residual stresses, surface defects, or work hardening that negatively affect fatigue performance and dimensional accuracy. Consequently, the sequence in which these processes are applied can critically determine the instrument's phase transformation behaviour, grain structure, surface integrity and overall mechanical properties. These factors, in turn, directly influence the instrument's performance, affecting its flexibility and resistance to fracture. Therefore, determining the optimal sequence of manufacturing steps is essential for achieving the desired material characteristics. The present study was designed to assess the impact of the sequence of manufacturing steps—specifically, the order of heat treatment and machining—on the metallurgical properties and mechanical performance of two versions of the same instrument. One version, Procodile Q, underwent heat treatment before machining, while the other, Procodile +HT, was heat‐treated after machining. The null hypothesis for this study states that the sequence of manufacturing steps (heat treatment before machining vs. heat treatment after machining) has no significant effect on the metallurgical properties or mechanical performance of tested instruments.

## 2 Material and Methods

This manuscript complies with the Preferred Reporting Items for Laboratory studies in Endodontology (PRILE) guidelines (Figure S1) and the respective checklist set forth by the Australian Endodontic Journal.

### 2.1 Sample Selection

**FIGURE 1:**
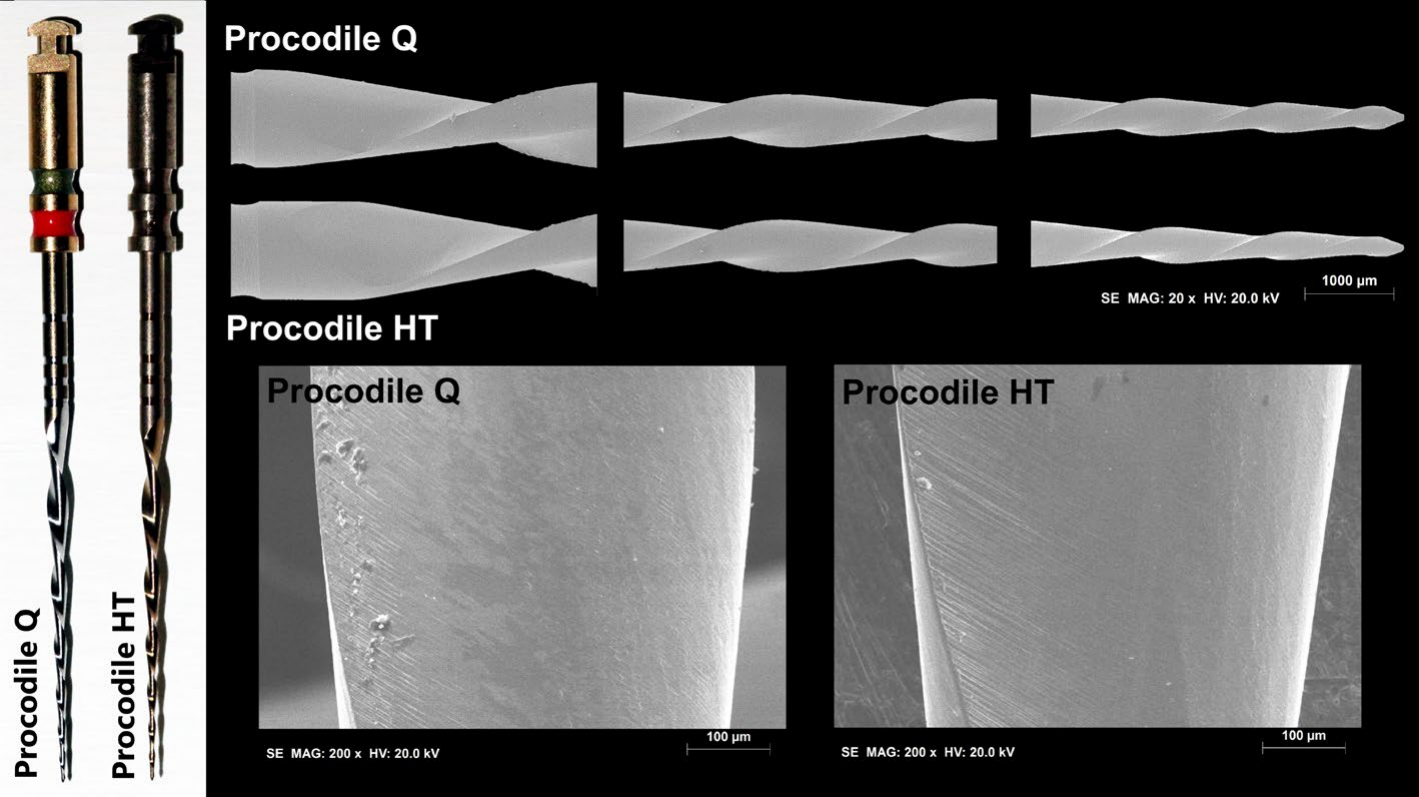
Macroscopic analysis of Procodile Q, instrument with heat treatment applied before machining, and Procodile +HT, instrument with heat treatment applied after machining (13.6× magnification). Design analysis revealed consistency and uniformity of both instruments. Scanning electron microscope inspection revealed similar characteristics between the groups, with both exhibiting symmetrical blade designs, non‐cutting tips and surface finishes marked by parallel lines from the manufacturing process. Only minimal surface irregularities and some residue near the cutting edge, likely resulting from the machining process, were observed at 20× and 200× magnifications.

Procodile Q (*n* = 74; Komet, Lot PA01704498) and the Procodile +HT prototype (*n* = 74; Komet, Lot PA01704491) reciprocating instruments, each 25 mm in length, with a tip size of 25 and a 0.06 taper, were compared in terms of their geometric design, metallurgical properties and mechanical performance. Prior to testing, all instruments underwent microscopic inspection at 13.6× magnification with LED illumination (Opmi Pico; Carl Zeiss Surgical, Jena, Germany) to detect any significant defects, such as blade design irregularities or unwinding that could disqualify them from the study. No defects were found, and all instruments were considered suitable for inclusion (Figure 1).

### 2.2 Design Assessment

Six instruments from each group were randomly selected and examined under a dental microscope (Opmi Pico, Carl Zeiss Surgical) at 13.6× magnification. A digital camera (Canon EOS 500D; Canon, Tokyo, Japan) captured images to assess the length of the active cutting blade, number of spirals, spirals per millimetre and spiral direction. The instruments were mounted on a file holder and examined under a scanning electron microscope (SEM) (S‐2400, Hitachi, Tokyo, Japan) at 20× and 200× magnifications to assess spiral geometry (symmetrical or asymmetrical), tip design (active or non‐active), surface markings from the manufacturing process and any minor defects or irregularities. Surface roughness was measured at the apical, middle and coronal thirds of the active blade of three instruments from each group using a New View 7100 Profilometer (Zygo Co, Middlefield, CT). This system measures surface heights from 0.1 to 1.0 mm, with a vertical resolution of 0.1 nm. Three measurements were taken per third in randomly selected areas, totalling nine measurements per instrument. Groove depth was calculated as the average of these nine measurements.

### 2.3 Metallurgical Characteristics

A semi‐quantitative elemental analysis was performed on three instruments from each group using energy‐dispersive X‐ray spectroscopy (EDS) with a standard SEM unit (DSM‐962, Carl Zeiss Microscopy GmbH) and an Inca X‐act EDS detector (Oxford Instruments NanoAnalysis, Abingdon, United Kingdom). The SEM operated at 20 kV and 3.1 A after a 10‐min vacuum process. Data were collected from a 500 μm × 500 μm area for 1 min at a 25 mm working distance. The ZAF correction method adjusted for atomic number (Z), absorption (A) and fluorescence (F) effects, ensuring accurate analysis [7]. The corrected data were processed using specialised software to determine the metallic element proportions (Microanalysis Suite v.4.14; Oxford Instruments NanoAnalysis, Abingdon, United Kingdom).

Differential scanning calorimetry (DSC) tests assessed phase transformation temperatures following specified guidelines [8]. This protocol ensures reliable measurement of thermal events, including martensitic and austenitic phase transitions, by monitoring heat flow changes with temperature. Small fragments (4–5 mm long, 5–10 mg) were cut from the active blade of each reference instrument and immersed in an etching solution (45% nitric acid, 25% hydrofluoric acid, 30% distilled water) for 2 min. After neutralising with distilled water, specimens were placed in an aluminium pan inside the DSC device, with an empty pan as the control. The heat cycle lasted 1 h and 40 min in a nitrogen gas atmosphere, with a temperature range from −150°C to 150°C and a 10°C per minute rate of increase. These procedures were performed in 3 instruments of each group. The DSC data and graphs were processed using Netzsch Proteus Thermal Analysis software (Netzsch‐Gerätebau GmbH, Selb, Germany).

### 2.4 Mechanical Performance

Eight mechanical parameters were evaluated to assess the performance of both instruments: time to fracture (at 20°C and 35°C), maximum torque, maximum rotation angle, bending strength, buckling strength, cutting efficiency and microhardness. The ideal sample size was calculated from six initial tests, using an alpha error of 0.05, power of 80% and effect sizes (±standard deviation) as follows: 115.3 (±61.7) for time to fracture at 20°C, 56.7 (±36.6) at 35°C, 4.5 (±17.4) for maximum torque, 0.02 (±0.09) for maximum rotation angle, 16.5 (±24.2) for bending strength, 1.6 (±14.0) for buckling strength, 1.4 (±13.4) for cutting efficiency and 15.0 (±29.9) for microhardness. The calculated required sample sizes were 6, 8, 318, 235, 36, 1202, 2819 and 64, respectively. Given the large sample sizes required for some parameters, which would likely yield statistically significant but limited practical or clinical relevance, the sample size calculation focused on time to fracture. A final sample size of 10 instruments was chosen to evaluate the time to fracture (at 20°C and 35°C), maximum torque, maximum rotation angle, bending strength and buckling strength. For microhardness, five indentations were performed on two instruments per group, in a total of 10 measurements.

The cyclic fatigue test was performed at room (20°C ± 1°C) and body temperature (35°C ± 1°C). To ensure consistent conditions, the cyclic fatigue device was submerged in a histology water bath (Leica HI1210, Leica Biosystems, UK) filled with distilled water, with continuous temperature monitoring. The instruments were mounted on a 6:1 reduction handpiece (Sirona Dental Systems GmbH, Bensheim, Germany), powered by a torque‐controlled motor set to the RECIPROC ALL mode (VDW Silver; VDW GmbH), whose exact speed and torque settings are proprietary and undisclosed by the manufacturer. They were operated in a fixed position within a stainless‐steel curved tube apparatus with a 6 mm radius and 86° angle. Fracture was determined by visual and auditory observation, and the time to fracture (in seconds) was recorded with a digital chronometer. Torsional strength, assessed by maximum torque (N·cm) and rotation angle (°), and bending resistance, determined by maximum bending load (gf), were evaluated according to an international standard [9]. Maximum buckling load (N) was measured using a universal testing machine with a 1 kN load cell (Instron Corporation 4502; series H3307) [10].

The cutting ability test used a custom apparatus connecting the handpiece of an endodontic motor (VDW Gold) to a 500 N load cell of a universal testing machine. Each instrument was positioned at the upper part of a simulated straight canal (size 15, taper 0.02) in a bone block model (PCF 10; Sawbones, Vashon, WA, USA). The test began when the machine applied a pre‐load of 10 gf. The instrument, set to RECIPROC ALL mode, moved forward 3 mm inside the canal, followed by a 2 mm backward movement, advancing 1 mm per cycle. This sequence repeated until the instrument advanced 10 mm. The recorded data included the maximum force, used to assess cutting ability; a higher force indicated lower cutting efficiency [11].

The microhardness evaluation created indentations on each instrument using a Vickers hardness tester (Duramin; Struers Inc., Cleveland, OH), following American Society for Testing and Materials guidelines [12]. All specimens were mounted on acrylic blocks. A total of 10 indentations were made, with 5 on each of 2 instruments per group. The indentations used a diamond indenter with a 100 gf load for 15 s [13]. Results were expressed as the Hardness Vickers Number (HVN) under ×40 magnification.

### 2.5 Statistical Analysis

Data normality was assessed using the Shapiro–Wilk test. Normally distributed data were reported as means with standard deviations, while non‐normally distributed data were reported as medians with interquartile ranges. Time to fracture, cutting ability, angle of rotation, buckling strength, microhardness and surface roughness were compared using the independent Student's t‐test. The nonparametric Mann–Whitney test (Wilcoxon Rank‐Sum Test) compared maximum torque to fracture and maximum bending load. A significance level of 5% was set for all statistical comparisons (SPSS v22.0 for Windows; SPSS Inc.).

## 3 Results

### 3.1 Design Assessment

**FIGURE 2:**
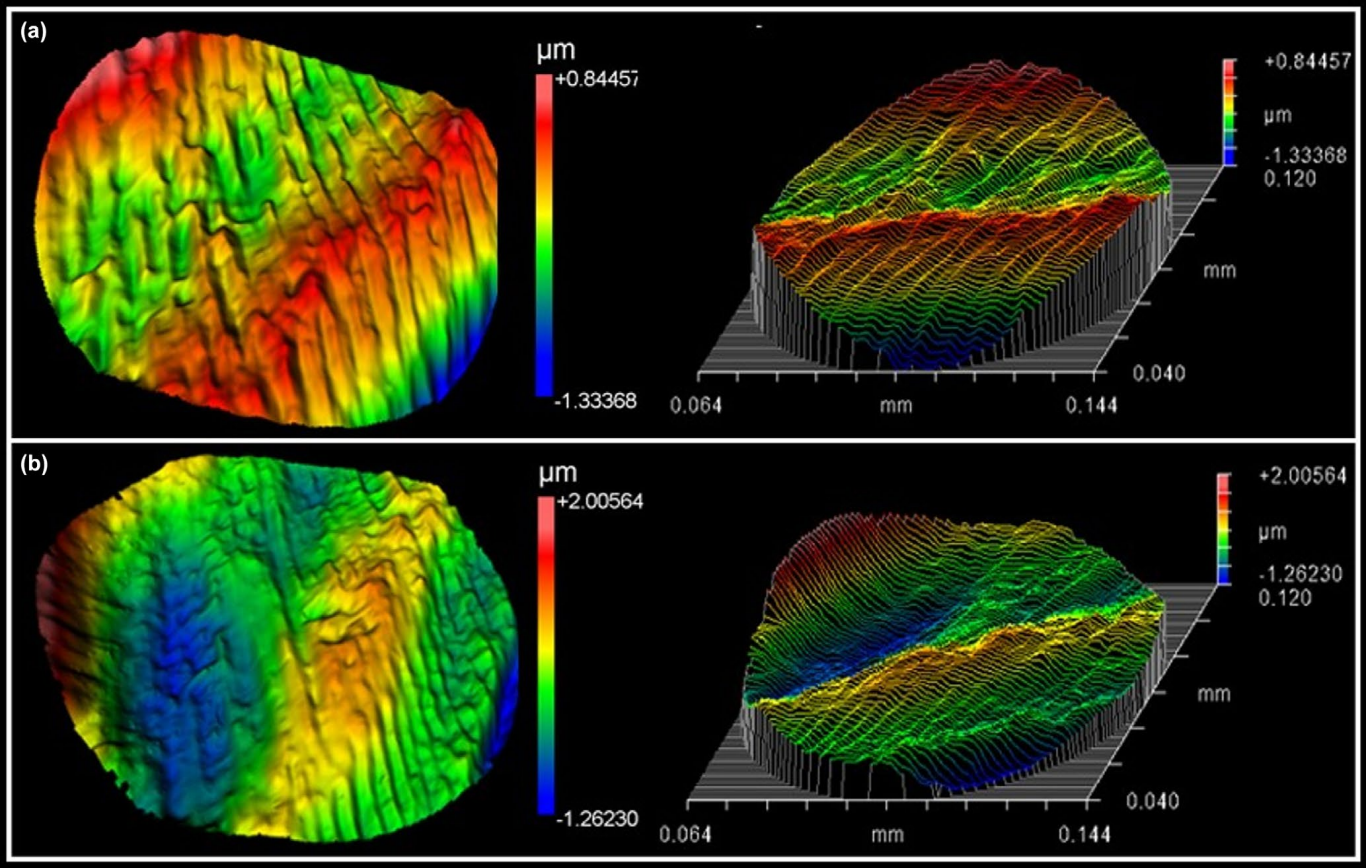
Surface roughness of (a) Procodile +HT and (b) Procodile Q instruments as measured using a profilometer. The colour scale represents surface height (in μm), with red indicating peaks and blue indicating valleys, corresponding to variations in surface roughness. The left images display top‐view colour maps, while the right images show the same data in 3D perspective plots with contour lines.

Design analysis showed consistency between the instruments. Procodile Q and Procodile +HT had similar characteristics, with an 18 mm active blade length and 7 spirals, corresponding to 0.39 spirals per millimetre. All spirals were oriented counterclockwise. SEM inspection confirmed both instruments had symmetrical blade designs, with no discontinuities or irregularities. The tips were non‐cutting, and the surface finishes showed parallel line markings from the manufacturing process (Figure 1). Procodile Q had higher surface roughness than Procodile +HT (*p* = 0.041) (Figure 2, Table 1).

**TABLE 1:** Mean (standard deviation) or median [interquartile range] results of time to fracture at 20°C and 35°C (in seconds), maximum torque (in N.cm), angle of rotation (in °), maximum bending load (in gf), buckling strength (in gf), cutting ability (in gf), microhardness (HVN) and surface roughness (RA) of tested instruments.

	Procodile Q	Procodile +HT	*p*
Time to fracture (20°C)	358 ± 20	239 ± 19	< 0.0001*
Time to fracture (35°C)	291 ± 22	226 ± 21	< 0.0001*
Maximum torque	1.2 [0.1]	1.2 [0.07]	0.256
Angle of rotation	464 ± 27	458 ± 20	0.605
Bending load	282 [27]	306 [37]	0.096
Buckling strength	298 ± 7	291 ± 25	0.475
Cutting ability	133 ± 17	130 ± 18	0.580
Microhardness	419 ± 31	404 ± 29	0.286
Roughness	0.335 ± 0.08	0.248 ± 0.08	0.041*

*Note:* * Represents statistically significant differences between Procodile Q and Procodile +HT instruments (*p* < 0.05).

### 3.2 Metallurgical Characteristics

**FIGURE 3:**
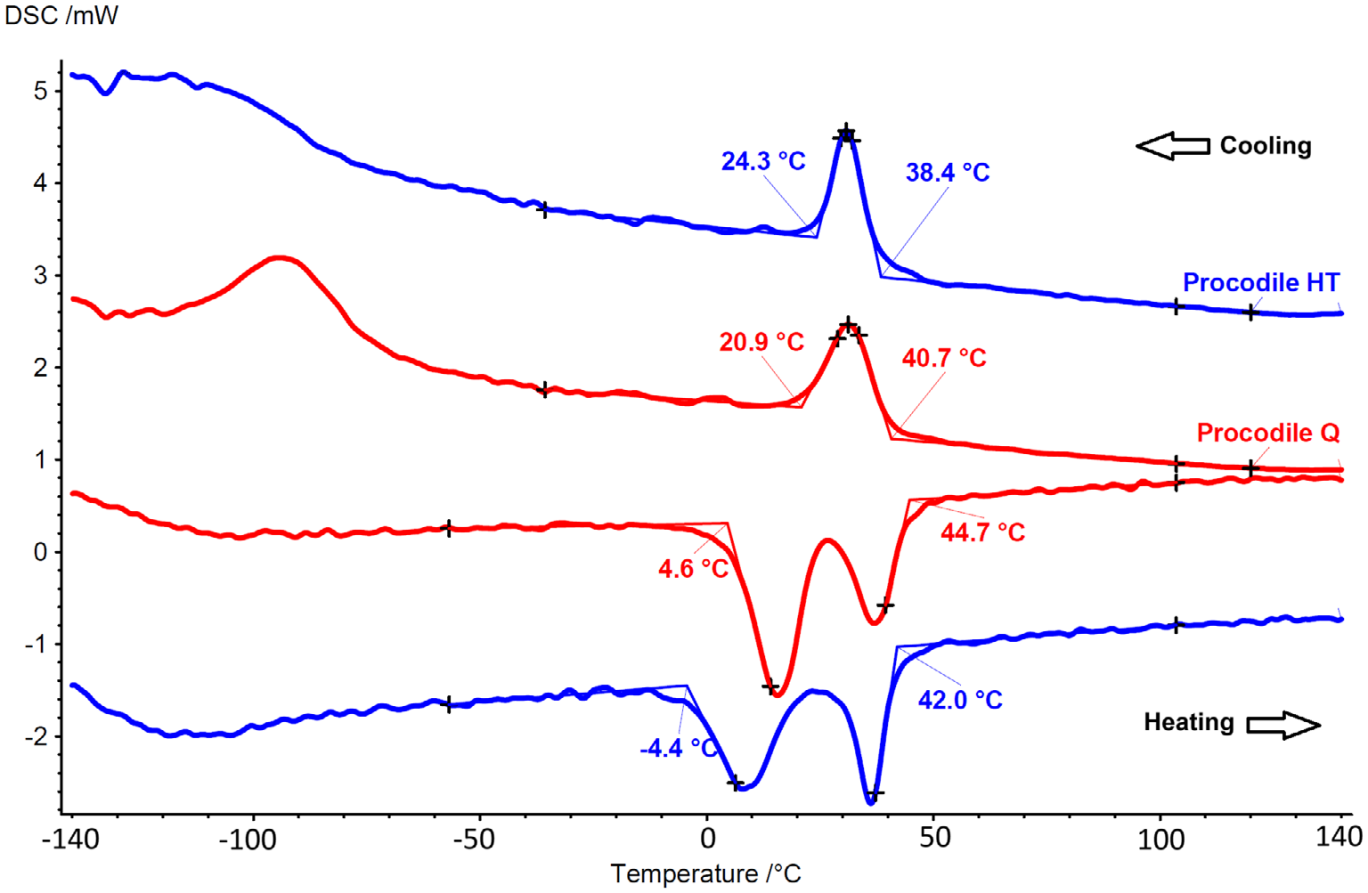
The DSC charts illustrate distinct phase transformation temperatures. Both instruments exhibit similar R‐phase start and finish temperatures during cooling, as well as austenitic start and finish temperatures during heating. The primary difference is observed where the transition from R‐phase to B19’ occurs between −70°C and −120°C in the Procodile Q instruments, while in the Procodile +HT, it begins around −80°C and completes above −150°C.

EDS analysis confirmed both alloys consisted of nickel and titanium, with no other metals detected. The elements were nearly equiatomic, with ratios of 1.017 for Procodile Q and 1.031 for Procodile +HT. In the DSC tests, both instruments exhibited R‐phase start and finish temperatures during cooling, around 40°C and 20°C, respectively. During heating, the austenitic start temperature was approximately 5°C for Procodile Q and −5°C for Procodile +HT, with both reaching the austenitic finish near 45°C. The main difference between the groups occurred during the R‐phase to B19’ transition in cooling. For Procodile Q, this transition happened between −70°C and −120°C, while for Procodile +HT, it started around −80°C and completed above −150°C (Figure 3). Additionally, Procodile Q showed a lower peak and broader range at its mid‐to‐high R‐phase position, suggesting post‐heat treatment hardening.

### 3.3 Mechanical Performance

Procodile Q showed superior cyclic fatigue resistance at both room (20°C ± 1°C) (*p* < 0.001) and body (35°C ± 1°C) (*p* < 0.001) temperatures compared to Procodile +HT. No significant differences were found between the two instruments in maximum bending load (*p* = 0.096), angle of rotation (*p* = 0.605), maximum torque (*p* = 0.256), buckling strength (*p* = 0.475), cutting ability (*p* = 0.580) and microhardness (*p* = 0.286) (Table 1).

## 4 Discussion

While many studies have examined the effects of different heat treatments and manufacturing processes on the performance of NiTi instruments [11, 13, 14, 15], none of them have specifically addressed how the sequence of these manufacturing steps—heat treatment versus machining—affects their properties. To conduct a meaningful investigation into this variable, it is essential to use instruments that are identical in every aspect, except for the order in which heat treatment and machining are applied. This approach ensures a controlled environment where the sole factor being tested is the sequence of the manufacturing process, allowing for a precise analysis of its impact on the instruments' performance. This study is the first to comprehensively assess how the sequence of manufacturing steps influences the metallurgical properties and mechanical performance of two versions of the same NiTi instrument (Figure 1). One version, Procodile Q, undergoes heat treatment before machining, while the other, Procodile +HT, a prototype with an identical geometric design, has heat treatment applied after machining. By isolating the effect of the manufacturing sequence, this research provides valuable insights into how the timing of heat treatment relative to machining can affect critical material properties and overall instrument performance. The sequence of machining and heat treatment had a minimal effect on the overall performance of NiTi instruments, with the primary differences observed in cyclic fatigue resistance and surface roughness. These findings led to the partial rejection of the null hypothesis, indicating that while the manufacturing sequence did influence certain specific properties, its impact on the overall functional performance of the instruments was limited.

NiTi alloys exhibit unique properties such as superelasticity and the shape memory effect, which are important for a wide range of applications [6, 16]. These properties are largely dictated by the alloy's microstructure, which is significantly influenced by heat treatment [6, 17], a process that modifies the phase transformation behaviour between martensitic and austenitic phases, directly affecting mechanical properties such as elasticity, strength and fatigue resistance [6]. For instance, controlled heat treatment can enhance flexibility by stabilising the martensitic phase or inducing an intermediate R‐phase [6, 18]. Additionally, heat treatment can improve fatigue resistance by minimising internal stresses and refining the grain structure, a key enhancement for applications like endodontic instruments subjected to cyclic loading [6]. However, improper or excessive heat treatment may lead to grain coarsening or the formation of secondary phases, which can degrade mechanical performance [17, 19]. Therefore, precise control over heat treatment parameters—including temperature, duration and cooling rate—is essential for optimising the mechanical properties and overall performance of NiTi alloys.

In addition to heat treatment, machining processes such as twisting, grinding, milling and electrical discharge machining (EDM) are frequently employed to shape NiTi components for various applications [6, 20, 21]. However, these processes can introduce residual stresses, surface defects and microstructural alterations that may undermine the mechanical properties of the alloy [20]. Surface integrity is particularly important in determining the fatigue life and fracture resistance of NiTi alloys, as poor machining techniques can lead to surface roughness and micro‐cracks, which act as stress concentrators, thereby reducing fatigue resistance [20]. Moreover, machining can locally modify the phase transformation temperatures, which can impact the superelastic and shape memory properties of the NiTi alloy [20, 22]. To mitigate these effects, it is essential to optimise machining parameters, including tool speed, feed rate and coolant application [20, 21, 22].

Work hardening, or strain hardening, is a phenomenon that commonly occurs during the machining of NiTi alloys. It results in increased material hardness and strength due to plastic deformation, which leads to a rise in dislocation density within the microstructure of the alloy [21, 23]. In the context of NiTi endodontic instruments, work hardening presents distinct challenges. The inherent superelasticity and shape memory properties of the NiTi alloy can complicate the machining process by accelerating tool wear, shortening tool lifespan and introducing residual stresses that negatively affect surface integrity and dimensional accuracy. Moreover, the hardened layer formed during machining becomes more resistant to cutting, thereby making material removal more difficult. To mitigate the effects of work hardening, it is crucial to optimise cutting speeds, use effective cooling methods and implement pre‐treatment processes [21, 22, 23].

Since both heat treatment and machining processes are recognised as critical factors influencing the mechanical properties of NiTi alloys [22], this study aimed to assess whether the sequence in which these processes are applied could impact the mechanical properties of NiTi endodontic instruments. When heat treatment is applied before machining, it can soften the material and reduce initial internal stresses, but subsequent machining may reintroduce localised surface stress and induce work hardening, a phenomenon characterised by increased localised strengthening of the alloy. Interestingly, in our study, Procodile Q (heat treatment before machining), which followed this sequence, exhibited significantly higher cyclic fatigue strength compared to Procodile +HT (machining before heat treatment). This finding may seem counterintuitive, as heat treatment applied after machining is generally assumed to relieve residual stress and optimise fatigue behaviour [22]. However, we hypothesise that controlled work hardening following a pre‐machining heat treatment may have conferred additional resistance to crack propagation, creating a more resilient microstructure even in the presence of slightly increased surface roughness (induced by the later machining process). This may suggest that the preliminary control of work hardening, if induced under favourable conditions, may help outweigh the surface integrity advantages conferred by post‐machining heat treatment. Thus, the observed superiority in fatigue strength may result from a complex interplay between heat treatment history and mechanical changes during manufacturing, highlighting the need to further explore how specific process sequences influence the lifespan longevity of NiTi instruments.

Interestingly, this superior performance cannot be attributed to surface roughness (the other parameter with statistical differences), as Procodile +HT demonstrated significantly lower roughness values (Table 1, Figure 3), likely due to the formation of a titanium oxide layer during heat treatment. This oxide layer can smooth the instrument's surface, leading to lower roughness values. Although reduced roughness is generally associated with improved fatigue performance by minimising stress concentration [24, 25], the enhanced fatigue resistance observed in Procodile Q suggests that other factors played a more prominent role. These findings suggest that factors beyond surface roughness—particularly those related to the sequence of manufacturing steps—may have played a more critical role in enhancing fatigue resistance. Specifically, the application of heat treatment prior to machining in Procodile Q could have led to beneficial metallurgical modifications, such as stabilisation of the martensitic or R‐phase structures, reduction in residual internal stresses, or improved control of grain boundary morphology. Additionally, optimised machining parameters following heat treatment may have preserved these metallurgical characteristics more effectively, contributing to enhanced mechanical performance. Thus, despite the higher surface roughness, the superior cyclic fatigue resistance observed in Procodile Q likely results from a synergistic effect between thermal processing and precise post‐treatment machining, which outweighs the influence of surface topography alone [26, 27].

Interestingly, no significant differences were observed in torsional and bending resistance between Procodile Q (heat treatment before machining) and Procodile +HT (machining before heat treatment). This could be attributed to the fact that these parameters primarily reflect the instrument's immediate response to applied forces, such as twisting or bending, which may not be as sensitive to the heat treatment or machining sequence as cyclic fatigue resistance. While cyclic fatigue is influenced by microstructural changes and stress accumulation over time, torsional and bending resistances are more dependent on the alloy composition and design. Additionally, the testing protocols for torsional and bending resistance may not have been sensitive enough to detect subtle differences between the two instruments. Thus, the similar results suggest that the manufacturing process may have a more significant impact on cyclic fatigue performance than on torsional and bending resistance, warranting further investigation into these effects.

Cyclic fatigue tests in this study were conducted at two distinct temperatures: room temperature (20°C ± 1°C) and body temperature (35°C ± 1°C). This dual‐temperature approach was selected because the R‐phase transformation finish temperature (Rf) of the tested instruments coincided precisely with 20°C, as determined by the DSC analysis (Figure 1). The R‐phase transformation occurs when the alloy transitions between martensitic and austenitic phases, and operating instruments near this temperature can introduce variability in mechanical behaviour. Even slight temperature fluctuations in this range could impact the alloy's flexibility and fatigue resistance, potentially affecting test accuracy. To mitigate this uncertainty, testing was also performed at 35°C, where the instruments are fully in their austenitic phase. This approach eliminated potential inconsistencies, providing a more accurate assessment of the instruments' performance under clinically relevant conditions. By testing at both temperatures, this study ensured robust and reliable data, highlighting any discrepancies linked to the temperature‐sensitive properties of NiTi alloys.

Several aspects of the cyclic fatigue test design merit clarification. The canal configuration used—a 86° curvature with a 6 mm radius—strikes a balance between clinical relevance and testing rigour. While the sharp curvature suggests a severe canal trajectory, the moderate radius produces a smoother, more extended curve that reduces overall severity without compromising the ability to distinguish differences in fatigue resistance. This configuration, widely adopted in previous studies [3, 10, 12, 14, 23, 24], serves as a standardised benchmark that enhances both reproducibility and comparability. The two instruments tested shared identical geometric and dimensional features, and all testing conditions were fully standardised. Consequently, observed differences in fatigue resistance are most likely attributable to metallurgical or manufacturing variations rather than canal anatomy. It is important to note that the goal of cyclic fatigue testing is not to predict an instrument's clinical lifespan, but to compare relative fatigue resistance under controlled and repeatable conditions. This enables researchers and clinicians to identify performance differences before moving to more complex and clinically variable models. Complementing this, the cutting ability test evaluated the instruments' performance in a simulated canal environment by measuring the force required as each file engaged the canal walls. Although the screw‐in effect is inherent to instrument motion, its influence was minimised through the use of a preload and controlled, stepwise advancement. This design allowed for a more accurate assessment of cutting efficiency, as higher recorded force generally corresponds to increased resistance and reduced efficiency. Unlike previous methods that focused primarily on lateral cutting, this approach offers a more comprehensive evaluation by capturing both cutting ability and resistance during advancement. Altogether, the results provide a realistic and clinically relevant assessment of instrument performance, offering valuable insights for informed decision‐making in endodontic practice.

A primary limitation of this study is that it focused on a single type of NiTi instrument, which may restrict the generalizability of the findings to other manufacturing processes or different NiTi instrument designs. Additionally, instruments with the specific manufacturing variations required for this study are not readily available on the market, and obtaining such specialised samples depends on the cooperation of the manufacturer, which can be challenging. As a result, further research involving a broader range of instruments is necessary to confirm the applicability of these findings across different NiTi alloys and manufacturing methods. Moreover, the absence of shaping and cleaning performance assessments in natural teeth represents an additional limitation. On the other hand, this study presents several significant strengths. The use of a multimethod approach, incorporating detailed analyses of geometric design, metallurgical characteristics and mechanical performance, enabled a thorough and robust evaluation of the instruments. By integrating various testing methods, the study provides a comprehensive understanding of the factors that influence instrument behaviour. Furthermore, the isolation of the heat treatment sequence–applied before or after machining–ensured that other major variables, such as commercial or design‐related factors, did not interfere with the analysis. This methodological rigour strengthens the reliability of the findings and contributes valuable evidence to the ongoing discourse on optimising the manufacturing processes for NiTi instruments.

## 5 Conclusions

The order of machining and heat treatment had minimal impact on the overall performance of NiTi instruments, notably affecting cyclic fatigue resistance and surface roughness. This suggests that although the manufacturing order may modify certain isolated features, its impact on the mechanical behaviour of the instrument appears to be limited.

## Author Contributions

**Emmanuel J. N. L. Silva:** conceptualization, analysis, experimental procedures, writing, review and editing (lead). **Jorge N. R. Martins:** conceptualization, analysis, experimental procedures, writing, review and editing (lead). **Livia Neri Menezes de Oliveira:** experimental procedures, writing. **Mylena do Rosário do Pereira:** experimental procedures. **Murilo Priori Alcalde:** experimental procedures, writing. **Victor T. L. Vieira:** experimental procedures, writing. **Francisco Manuel Braz Fernandes:** experimental procedures. **Marco A. Versiani:** conceptualization, analysis, experimental procedures, writing, review and editing (lead).

## Ethics Statement

The authors have nothing to report.

## Conflicts of Interest

The authors declare no conflicts of interest.

## Supporting information


**Figure S1.** PRILE flowchart.

## Data Availability

The data that support the findings of this study are available from the corresponding author upon reasonable request.
