# Supplementary material for: Manufacturing Order Matters? Exploring the Impact of Heat Treatment and Machining Sequences on the NiTi Instruments Properties
Source: Aust Endod J. 2025 May 26;51(2):390–7. doi: 10.1111/aej.12953 (PMC12351088; doi:10.1111/aej.12953)
Supplement: Supplementary file 1 — Figure S1. PRILE flowchart. [file AEJ-51-390-s001.pdf]

PRILE 2021 Flowchart

While heat treatments have unquestionably enhanced the properties of NiTi instruments, the specific sequence of manufacturing steps, particularly the order in which heat treatment and machining processes are applied, may play an important role in determining the final metallurgical and mechanical characteristics of these instruments. However, this aspect remains underexplored, leaving a gap on how these processes influence the performance and properties of NiTi instruments.

This study aimed to assess the impact of the sequence of manufacturing steps on the metallurgical properties and mechanical performance of two versions of the same nickel-titanium (NiTi) instrument, one with heat treatment applied before machining (Procodile Q) and the other with heat treatment applied after machining (Procodile +HT).

Ethics Committe Approval protocol: NA

NiTi reciprocating instruments: Procodile Q and Procodile +HT

Procodile Q (n=74) and the Procodile +HT (n=74) size 25/.06 reciprocating instruments

Design (length of the active cutting blade, number of spirals, spiral geometry, spirals direction, tip design, surface finishing, and surface roughness), metallurgical properties (nickel and titanium composition and phase transformation temperatures), and mechanical properties (time to fracture at 20 ºC and 35 ºC, maximum torque, maximum rotation angle, bending strength, buckling strength, cutting efficiency, and microhardness).

Scanning electron microscopy, profilometer, energy-dispersive X-ray spectroscopy, differential scanning calorimetry, mechanical tests.

The design analysis revealed consistency and uniformity between both instruments, which were composed of NiTi alloy in nearly equiatomic proportions. Slight differences were observed in the phase transformation temperatures. Procodile Q demonstrated superior cyclic fatigue resistance at both room and body temperatures ($P<0.001$), as well as higher surface roughness ($P=0.041$), compared to Procodile +HT. No significant differences were found between the two instrument types in the other tests ($p<0.05$).

The order of machining and heat treatment had minimal impact on the overall performance of NiTi instruments, notably affecting cyclic fatigue resistance and surface roughness, suggesting that while the manufacturing sequence may influence specific characteristics, its overall effect on clinical performance may remain limited.

This study was partially funded by XXX.

The authors deny any conflicts of interest related to this study.

***From: Nagendrababu V, Murray PE, Ordinola-Zapata R, Peters OA, Rôças IN, Siqueira JF Jr, Priya E, Jayaraman J, Pulikkotil SJ, Camilleri J, Boutsioukis C, Rossi-Fedele G, Dummer PMH (2021) PRILE 2021 guidelines for reporting laboratory studies in Endodontology: a consensus-based development. *International Endodontic Journal* May 3. doi: 10.1111/iej.13542. https://onlinelibrary.wiley.com/doi/abs/10.1111/iej.13542.**

For further details visit: http://pride-endodonticguidelines.org/prile
